# Association between abdominal obesity indices and risk of cardiovascular events in Chinese populations with type 2 diabetes: a prospective cohort study

**DOI:** 10.1186/s12933-022-01670-x

**Published:** 2022-11-01

**Authors:** Tingting Qiao, Tao Luo, Hualian Pei, Bahegu Yimingniyazi, Dilihumaer Aili, Aliya Aimudula, Hui Zhao, Huanwen Zhang, Jianghong Dai, Duolao Wang

**Affiliations:** 1 Department of Epidemiology and Biostatistics, School of Public Health, Xinjiang Medical University, Urumqi, 830017 China; 2 Department of Clinical Nursing, School of Nursing, Xinjiang Medical University, Urumqi, 830017 China; 3 Department of Nursing, Ningbo First Hospital, Ningbo, 315012 China; 4 Department of Clinical Sciences, Liverpool School of Tropical Medicine, Liverpool, L3 5QA UK

**Keywords:** Type 2 diabetes, Abdominal obesity, Cardiovascular disease, Visceral adiposity index, Lipid accumulation product, Chinese visceral adiposity index

## Abstract

**Background:**

Waist circumference (WC), visceral adiposity index (VAI), lipid accumulation product (LAP), and Chinese visceral adiposity index (CVAI) are considered surrogate indicators of abdominal fat deposition, but the longitudinal association of these indices with cardiovascular (CV) events in adults with type 2 diabetes (T2D) remains unclear. Our study aimed to examine the associations between abdominal obesity indices and incident CV events among people with T2D and to compare their predictive performance in risk assessment.

**Methods:**

The present study included 2328 individuals with T2D from the Xinjiang Multi-Ethnic Cohort. Multivariable Cox regression analyses were applied to assess the associations between abdominal obesity indices and CV events. Harrell's concordance statistic (C-statistic), net reclassification improvement (NRI) index, and integrated discrimination improvement (IDI) index were utilized to evaluate the predictive performance of each abdominal obesity index.

**Results:**

At a median follow-up period of 59 months, 289 participants experienced CV events. After multivariable adjustment, each 1-SD increase in WC, VAI, LAP, and CVAI was associated with a higher risk of CV events in people with T2D, with adjusted hazard ratios (HRs) being 1.57 [95% CI (confidence interval): 1.39–1.78], 1.11 (95% CI 1.06–1.16), 1.46 (95% CI 1.36–1.57), and 1.78 (95% CI 1.57–2.01), respectively. In subgroup analyses, these positive associations appeared to be stronger among participants with body mass index (BMI) < 25 kg/m^2^ compared to overweight/obese participants. As for the predictive performance, CVAI had the largest C-statistic (0.700, 95% CI 0.672–0.728) compared to VAI, LAP, WC, and BMI (C-statistic: 0.535 to 0.670, all *P* for comparison < 0.05). When the abdominal obesity index was added to the basic risk model, the CVAI index also showed the greatest incremental risk stratification (C-statistic: 0.751 vs. 0.701, *P* < 0.001; IDI: 4.3%, *P* < 0.001; NRI: 26.6%, *P* < 0.001).

**Conclusions:**

This study provided additional evidence that all abdominal obesity indices were associated with the risk of CV events and highlighted that CVAI might be a valuable abdominal obesity indicator for identifying the high risk of CV events in Chinese populations with T2D. These results suggest that proactive assessment of abdominal obesity could be helpful for the effective clinical management of the diabetic population.

**Supplementary Information:**

The online version contains supplementary material available at 10.1186/s12933-022-01670-x.

## Background

The International Diabetes Federation published a report in 2021 indicating that there are about 536 million people with diabetes globally, while China has 140 million diabetic patients, ranking first in the world [1]. Compared to those without type 2 diabetes (T2D), adults with T2D are at a twofold higher risk of incident cardiovascular disease (CVD), which occurs earlier and more severely [2, 3]. At the same time, the primary cause of hospital admissions and death in people with T2D is CVD, which poses a heavy economic healthcare burden [4]. A meta-analysis estimated that CVD impacted 32.2% of individuals with T2D and caused 9.9% of deaths in T2D patients (accounting for 50.3% of all deaths) [5]. Thus, it is vital to identify modifiable CVD risk factors among individuals with T2D to provide effective prevention strategies.

Obesity has been established as a crucial risk factor for cardiometabolic disease and premature death [6]. Body mass index (BMI) is frequently utilized to classify overweight or obesity, but the evidence from published studies on the relationship between BMI and CVD risk in people with T2D is inconsistent. Several studies indicated that BMI was positively associated with incident cardiovascular (CV) events among people with T2D [7–9], but others showed a negative [10, 11] or no association [12, 13]. Such inconsistent results suggest that BMI is not a perfect estimator of obesity, as it neither distinguishes between lean mass and total fat mass nor captures the distribution of body fat [14, 15]. Recent evidence demonstrated that adipose tissue distribution rather than overall adiposity is a significant factor in determining CVD risk [16]. Moreover, increasing studies have shown that abdominal fat deposition plays a crucial role in cardiometabolic diseases [16–18].

Radiological imaging techniques can accurately assess abdominal adiposity, but they are inappropriate for widespread epidemiological investigations due to their long duration, high price, and radiation hazards [19]. Hence, there is a need to establish reliable and easily accessible indices to evaluate abdominal adiposity. Waist circumference (WC) is a widely used indicator for abdominal obesity, but it has deficiencies in discriminating between subcutaneous and visceral adipose tissue. Several novel abdominal obesity indices established by combining anthropometric and lipid parameters were considered superior to traditional anthropometric parameters [20, 21]. These novel indices are the visceral adiposity index (VAI), lipid accumulation product (LAP), and Chinese visceral adiposity index (CVAI). Although several studies have suggested that these abdominal obesity indices could predict the occurrence of CVD in the general population [22–24], this issue is rarely examined in the T2D population. To date, only one cross-sectional study has revealed the relationship between abdominal obesity indicators and macrovascular complications of diabetes [25]. However, there is still unclear about the longitudinal association of these indices with CVD risk in people with T2D. In addition, although several studies have estimated and compared the predictive performances of abdominal obesity indices for chronic diseases in different populations [26–28], the findings were inconsistent. Thus, it is necessary to examine further which abdominal obesity index is the optimal predictor of CV events in people with T2D.

In this background, we explored the relationship between baseline abdominal obesity indices (WC, LAP, VAI, and CVAI) and incident CV events among people with T2D and compared their risk prediction performances.

## Methods

### Study design and participants

The Xinjiang Multi-Ethnic Cohort (XMC) is a longitudinal, population-based study in which participants were recruited from community health centers in three regions of Xinjiang (Urumqi, Hotan, and Yili), China. In brief, the XMC study was designed to explore the influences of genetics, environmental exposures, and lifestyle on health outcomes. Details of the XMC have been presented in a previous paper [29].

**Fig.1:**
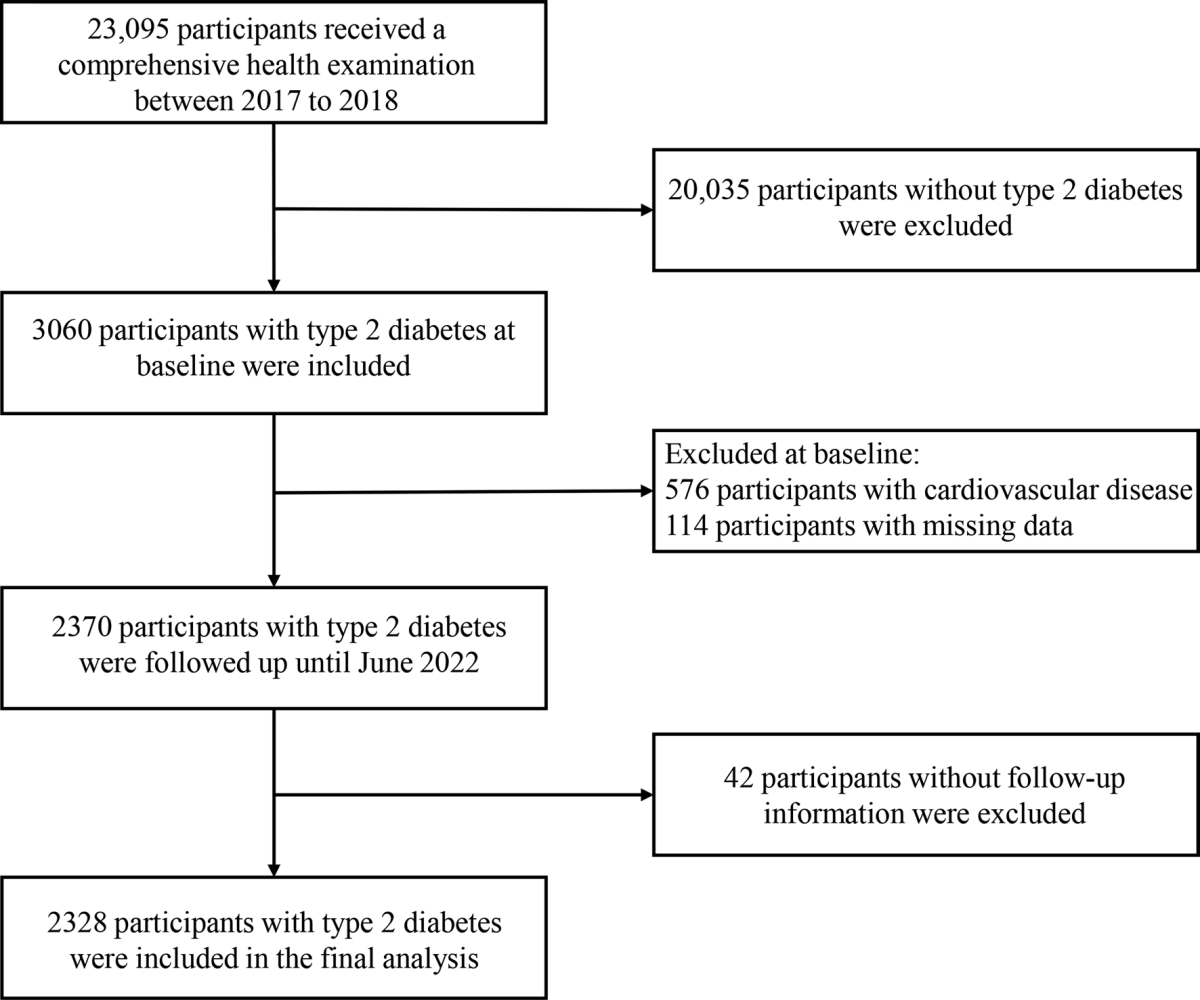
Flowchart of the study population

Between 2017 to 2018, 23,095 participants received a comprehensive health examination, and 3060 were identified as suffering from T2D. T2D was defined as any one of the following standards: (1) fasting blood glucose (FBG) level ≥ 7 mmol/L; (2) taking glucose-lowering agents; (3) previously diagnosed T2D by physicians. We excluded participants with a history of CVD (*n* = 576) at baseline, with incomplete data (*n* = 114), or without any follow-up information (*n* = 42). Ultimately, 2328 participants with T2D could be included in the present study (Fig. 1). Approval for this study protocol was granted by the Institutional Review Board of the Traditional Chinese Medical Hospital of Xinjiang Uygur Autonomous Region (2018XE0108). All participants signed an informed consent document.

### Baseline data collection and definitions

Demographic characteristics (age, gender, ethnicity, location, and education), medical history, use of antidiabetic agents, and lifestyle factors were collected by trained clinic staff through a standardized questionnaire. The ethnicity was classified as Han, Hui, Uyghur, and others (Kazakh, Mongolian, and Tibetan). Education status was categorized as primary school or below, middle school, and high school or further education. The location of residence was categorized as urban and rural. Smoking and drinking habits were classified as current and never or previous. Physical activity was defined as exercise more than once a week [30].

Trained physicians or nurses performed anthropometric measurements following standardized protocols. WC was checked at the middle point between the iliac crest and the lower rib margin. Weight and height were checked using an auto-anthropometer (SK-X80, China) with an accuracy of 0.1 kg and 0.1 cm, respectively. When participants were seated after a 10-min rest, blood pressure was assessed two times with a calibrated mercury sphygmomanometer, and the mean of the two assessments was applied for subsequent analysis. After an overnight fast, nurses collected participants' venous blood samples for laboratory tests. Triglycerides (TG), total cholesterol (TC), high-density lipoprotein cholesterol (HDL-C), low-density lipoprotein cholesterol (LDL-C), and FBG levels were performed in the laboratories of the community health centers with automated analyzers (Hitachi, Tokyo, Japan).

The formulas for BMI, VAI [31], LAP [32], and CVAI [21] were:

$${\text{BMI = weight}}\left( {{\text{kg}}} \right){\text{/height}}^{{2}} \left( {\text{m}} \right)$$

$${\text{VAI}}\;\left( {{\text{male}}} \right) = \left[ {\frac{{{\text{WC}}\left( {{\text{cm}}} \right)}}{{39.68 + 1.88 \times BMI\left( {{{kg} \mathord{\left/ {\vphantom {{kg} {m^{2} }}} \right. \kern-\nulldelimiterspace} {m^{2} }}} \right)}}} \right] \times \left[ {\frac{{{\text{TG}}\left( {{{{\text{mmol}}} \mathord{\left/ {\vphantom {{{\text{mmol}}} {\text{L}}}} \right. \kern-\nulldelimiterspace} {\text{L}}}} \right)}}{1.03}} \right] \times \left[ {\frac{1.31}{{HDL\left( {{{{\text{mmol}}} \mathord{\left/ {\vphantom {{{\text{mmol}}} {\text{L}}}} \right. \kern-\nulldelimiterspace} {\text{L}}}} \right)}}} \right]$$

$${\text{VAI}}\;\left( {{\text{female}}} \right) \, = \;\left[ {\frac{{\text{WC(cm)}}}{{36.58 + 1.89 \times {\text{BMI(kg}}/{\text{m}}^{{2}} )}}} \right] \times \left[ {\frac{{TG({\text{mmol}}/{\text{L}})}}{0.81}} \right] \times \left[ {\frac{1.52}{{{\text{HDL}}({\text{mmol}}/{\text{L}})}}} \right]$$

$${\text{LAP }}\left( {{\text{male}}} \right)\; = \;\left[ {{\text{WC(cm)}} - 65} \right] \times {\text{TG(mmol}}/{\text{L}})$$

$${\text{LAP}}\;\left( {{\text{female}}} \right) \, = \;\left[ {{\text{WC(cm)}} - 58} \right] \times {\text{TG}}({\text{mmol}}/{\text{L}})$$

$${\text{CVAI }}\left( {{\text{male}}} \right) \, = \; - 267.93 + 0.68 \times {\text{age}}\left( {{\text{years}}} \right) + 0.03 \times {\text{BMI}}\left( {{\text{kg}}/{\text{m}}^{{2}} } \right) + 4.00 \times {\text{WC}}\left( {{\text{cm}}} \right) + 22.00 \times {\text{Lg TG}}\left( {{\text{mmol}}/{\text{L}}} \right){-}16.32 \times {\text{HDL}} ({\text{mmol}}/{\text{L}})$$

$${\text{CVAI }}\left( {{\text{female}}} \right) \, = \; - 187.32 + 1.71 \times {\text{age}}\left( {{\text{years}}} \right) + 4.32 \times {\text{BMI}}\left( {{\text{kg}}/{\text{m}}^{{2}} } \right) + 1.12 \times {\text{WC}}\left( {{\text{cm}}} \right) + 39.76 \times {\text{Lg TG}}\left( {{\text{mmol}}/{\text{L}}} \right){-}11.66 \times {\text{HDL}} ({\text{mmol}}/{\text{L}})$$

### Follow-up and outcomes assessment

Participants were followed up annually through health examinations and telephone interviews. In addition, Medical Record Information System, Medical Insurance System, Chronic Disease Management System, and Death Information Registration System were used to confirm health outcomes further. The primary endpoint was the time in days from admission to the study to the first occurrence of a CV event, including nonfatal myocardial infarction, nonfatal stroke, or CV death (fatal myocardial infarction, fatal stroke, and other CV death) [33]. Participants were tracked from baseline to date of death or CV events or June 2022, whichever occurred first.

### Statistical analysis

Data were summarized as percentages, mean ± standard deviation (SD), or median (interquartile range) where appropriate. When comparing the differences between the two groups, the Chi-square test was performed for categorical data, and the Student's *t*-test or Mann-Whitney U-test was for continuous data. Kaplan–Meier curves were plotted to describe the cumulative rates of CV events by groups according to the quartiles of abdominal obesity indices and were compared using the log-rank test. Cox proportional-hazards regression analysis was utilized to obtain the hazard ratios (HRs) of CV events across the quartiles of the WC, VAI, LAP, and CVAI. We also evaluated the risk of CV events associated with one SD increase in abdominal obesity indices. Model 1 was unadjusted. Model 2 was adjusted for gender, age, ethnicity, education, smoking status, and drinking status. Then, the third model additionally adjusted for LDL-C, TC, FBG, systolic blood pressure, diastolic blood pressure, physical activity, antidiabetic agents, and diabetes duration. Furthermore, the restricted cubic spline analysis was conducted to examine the non-linear relationships between abdominal obesity indices and hazards of incident CV events. Subgroup analyses were conducted based on age, BMI, ethnicity, and usage of antidiabetic agents, and interactions between subgroups were examined.

Harrell's concordance statistic (C-statistic) was calculated to estimate the predictive performance of BMI, WC, VAI, LAP, and CVAI for incident CV events in people with T2D. Differences between the C-statistics of these abdominal obesity indices were compared using the DeLong test [34]. Also, the net reclassification improvement (NRI) index and integrated discrimination improvement (IDI) index were calculated to estimate the incremental predictive value of WC, VAI, LAP, and CVAI beyond the established risk factors for CV events. Considering that non-CVD-related death is a competing risk, we performed a sensitivity analysis by using Fine–Gray proportional sub-distribution hazards models to assess the robustness of associations between abdominal obesity indices and CV events. Moreover, we included participants who were excluded because of missing data and repeated the primary analyses to assess the robustness of the results. Multiple imputation by chained equations was performed to fill the missing covariates at baseline. Statistical analyses were done with STATA MP version 17.0 (Stata Corp) and R version 4.2.1. *P* value < 0.05 was considered statistical significance.

## Results

### Baseline characteristics

In total, 2328 participants (male, *n* = 976; female, *n* = 1352) with T2D were available for analysis, and the mean age was 59.30 ± 10.01 years at baseline. At a median follow-up of 59 months (interquartile range: 56–61), 289 (12.41%) participants experienced CV events (including 135 strokes and 176 myocardial infarctions), and 70 (3.01%) participants died. The baseline characteristics of participants by CVD status at follow-up are summarized in Table 1. Compared to those without CVD, individuals who developed CVD tended to be older, current smokers and drinkers, and less physically active. Moreover, they also had higher systolic blood pressure, diastolic blood pressure, BMI, LDL-C, TG, WC, VAI, LAP, and CVAI and lower HDL-C than those without CVD (all *P*-values < 0.05, Table 1).

**Table 1:** Baseline characteristics of participants according to the cardiovascular disease status at follow-up

Variables	Total (*n* = 2328)	Incident CVD (*n* = 289)	Non-case (*n* = 2039)	*P-*value
Age, years	59.30 ± 10.01	65.58 ± 7.47	58.41 ± 10.00	< 0.001
Diabetes duration, years	7.73 ± 3.17	7.94 ± 2.84	7.70 ± 3.21	0.227
Gender, *n* (%)				0.915
Male	976 (41.92)	122 (42.21)	854 (41.88)	
Female	1352 (58.08)	167 (57.79)	1185 (58.12)	
Ethnicity, *n* (%)				0.072
Han	1015 (43.60)	128 (44.29)	887 (43.50)	
Hui	324 (13.92)	29 (10.03)	295 (14.47)	
Uyghur	901 (38.70)	125 (43.25)	776 (38.06)	
Others	88 (3.78)	7 (2.42)	81 (3.97)	
Education, *n* (%)				0.098
Primary school or below	1508 (64.78)	203 (70.24)	1305 (64.00)	
Middle school	548 (23.54)	60 (20.76)	488 (23.93)	
High school or further	272 (11.68)	26 (9.00)	246 (12.06)	
Location, *n* (%)				
Urban	1045 (44.89)	136 (47.06)	909 (44.58)	0.428
Rural	1283 (55.11)	153 (52.94)	1130 (55.42)	
Current smoking, *n* (%)	337 (14.48)	62 (21.45)	275 (13.49)	< 0.001
Current drinking, *n* (%)	224 (9.62)	39 (13.49)	185 (9.07)	0.017
Physical activity, *n* (%)	676 (29.04)	69 (23.88)	607 (29.77)	0.039
Antidiabetic agents, *n* (%)	1196 (51.37)	162 (56.06)	1034 (50.71)	0.089
SBP, mmHg	133.47 ± 18.16	137.75 ± 20.42	132.86 ± 17.74	< 0.001
DBP, mmHg	79.75 ± 10.84	81.43 ± 11.75	79.51 ± 10.69	0.005
Total cholesterol, mmol/L	4.91 ± 2.28	5.14 ± 2.63	4.87 ± 2.23	0.059
HDL cholesterol, mmol/L	1.44 ± 0.57	1.32 ± 0.45	1.45 ± 0.59	< 0.001
LDL cholesterol, mmol/L	2.69 ± 1.02	2.80 ± 0.82	2.68 ± 1.04	0.044
Triglyceride, mmol/L	1.44 (1.04, 1.95)	1.87 (1.24, 2.39)	1.40 (1.00, 1.85)	< 0.001
FBG, mmol/L	7.28 ± 3.21	7.50 ± 3.25	7.25 ± 3.20	0.221
Body mass index, kg/m^2^	26.42 ± 3.73	26.86 ± 3.81	26.36 ± 3.71	0.034
Waist circumference, cm	91.18 ± 10.95	95.76 ± 10.23	90.53 ± 10.90	< 0.001
VAI	1.76 (1.14, 2.66)	2.42 (1.64, 3.37)	1.67 (1.08, 2.54)	< 0.001
LAP	41.15 (26.00, 65.01)	60.20 (39.60, 89.10)	38.72 (24.70, 60.72)	< 0.001
CVAI	121.08 ± 39.57	145.52 ± 33.89	117.61 ± 39.10	< 0.001

Data are summarized as number (percentage), mean ± standard deviation, or median (interquartile range)

*CVD* cardiovascular disease, *SBP* systolic blood pressure, *DBP* diastolic blood pressure, *HDL* high-density lipoprotein, *LDL* low-density lipoprotein, *FBG* fasting blood glucose, *VAI* visceral adiposity index, *LAP* lipid accumulation product, *CVAI* Chinese visceral adiposity index

### Relationship between abdominal obesity indices and cardiovascular events among people with type 2 diabetes

**Fig.2:**
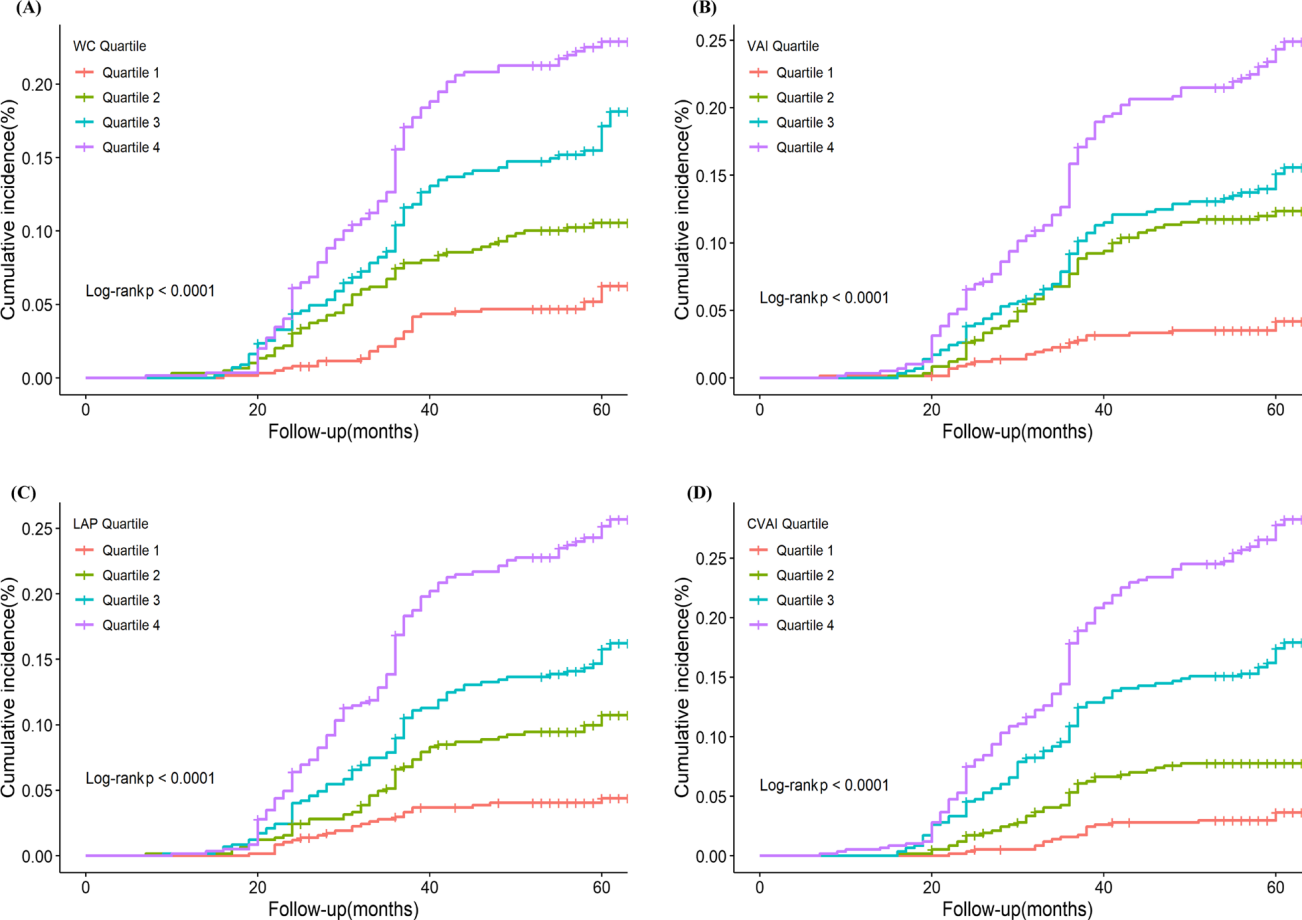
Kaplan–Meier incidence rate of cardiovascular events in people with type 2 diabetes according to quartiles of abdominal obesity indices. **A** waist circumference (WC), **B** visceral adiposity index (VAI), **C** lipid accumulation product (LAP), and **D** Chinese visceral adiposity index (CVAI)

Kaplan–Meier curves for incident CV events among people with T2D by quartiles of abdominal obesity indices are presented in Fig. 2. Higher baseline WC, VAI, LAP, and CVAI were correlated with an elevated risk of CV events in people with T2D (all log-rank tests *P* < 0.001).

The Cox regression models were applied to investigate the association of abdominal obesity indices with the risk of CV events among people with T2D (Table 2). Compared to the lowest quartile group of baseline WC, VAI, LAP, and CVAI, the fully adjusted HRs (95%) for CV events in the highest quartile group were 3.92 (95% CI 2.62–5.88), 6.88 (95% CI 4.27–11.07), 6.03 (95% CI 3.85–9.47), and 5.67 (95% CI 3.47–9.27), respectively. Such associations remained significant when all abdominal obesity indices were estimated as continuous variables. After adjusting for all covariates, each 1-SD increase in WC, VAI, LAP, and CVAI was associated with a higher risk of CV events among people with T2D, with adjusted HR being 1.57 (95% CI 1.39–1.78), 1.11 (95% CI 1.06–1.16), 1.46 (95% CI 1.36–1.57), and 1.78 (95% CI 1.57–2.01), respectively. Furthermore, restricted cubic spline regression revealed the non-linear relationships between abdominal obesity indices and hazards of incident CV events in people with T2D (Additional file 1: Fig. S1).

**Table 2:** Association between baseline abdominal obesity indices and incident cardiovascular events in people with type 2 diabetes

Indices	Event/ Total	Model 1		Model 2		Model 3	
		*HR* (95% CI)	*P*-value	*HR* (95% CI)	*P*-value	*HR* (95% CI)	*P*-value
WC							
Per 1 SD increase	289/2328	1.52 (1.37–1.69)	< 0.001	1.59 (1.41–1.80)	< 0.001	1.57(1.39–1.78)	< 0.001
Quartiles							
Q1(≤ 84.10)	33/614	1.00		1.00		1.00	
Q2(84.20–90.00)	59/599	1.89 (1.23–2.89)	0.003	1.75 (1.14–2.68)	0.010	1.72 (1.12–2.65)	0.013
Q3(90.10–98.00)	85/559	2.99 (2.00–4.47)	< 0.001	2.95 (1.97–4.43)	< 0.001	2.88 (1.92–4.32)	< 0.001
Q4(> 98.00)	112/556	4.05 (2.75–5.97)	< 0.001	4.10 (2.74–6.12)	< 0.001	3.92 (2.62–5.88)	< 0.001
VAI							
Per 1 SD increase	289/2328	1.09 (1.05–1.13)	< 0.001	1.11 (1.07–1.16)	< 0.001	1.11 (1.06–1.16)	< 0.001
Quartiles							
Q1(≤ 1.14)	22/582	1.00		1.00		1.00	
Q2(1.15–1.76)	66/582	3.11 (1.92–5.04)	< 0.001	3.09 (1.90–5.02)	< 0.001	3.03 (1.86–4.93)	< 0.001
Q3(1.77–2.66)	79/582	3.77 (2.35–6.04)	< 0.001	4.10 (2.54–6.64)	< 0.001	3.97 (2.45–6.44)	< 0.001
Q4(> 2.66)	122/582	6.11 (3.88–9.62)	< 0.001	7.14 (4.46–11.41)	< 0.001	6.88 (4.27–11.07)	< 0.001
LAP							
Per 1 SD increase	289/2328	1.41 (1.32–1.50)	< 0.001	1.47 (1.37–1.57)	< 0.001	1.46 (1.36–1.57)	< 0.001
Quartiles							
Q1(≤ 26.00)	24/583	1.00		1.00		1.00	
Q2(26.01–41.15)	56/581	2.40 (1.49–3.88)	< 0.001	2.51 (1.55–4.06)	< 0.001	2.50 (1.54–4.04)	< 0.001
Q3(41.16–65.00)	82/582	3.58 (2.27–5.64)	< 0.001	3.71 (2.35–5.88)	< 0.001	3.65 (2.30–5.80)	< 0.001
Q4(> 65.00)	127/582	5.84 (3.78–9.04)	< 0.001	6.18 (3.96–9.63)	< 0.001	6.03 (3.85–9.47)	< 0.001
CVAI							
Per 1 SD increase	289/2328	1.87 (1.68–2.08)	< 0.001	1.80 (1.60–2.04)	< 0.001	1.78 (1.57–2.01)	< 0.001
Quartiles							
Q1(≤ 96.58)	19/582	1.00		1.00		1.00	
Q2(96.59–121.59)	43/582	2.34 (1.37–4.02)	0.002	1.88 (1.09–3.24)	0.023	1.91 (1.11–3.30)	0.020
Q3(121.60–146.38)	89/582	5.05 (3.08–8.29)	< 0.001	3.81 (2.31–6.28)	< 0.001	3.71 (2.24–6.15)	< 0.001
Q4(> 146.38)	138/582	8.14 (5.04–13.15)	< 0.001	5.86 (3.61–9.53)	< 0.001	5.67 (3.47–9.27)	< 0.001

Model 1: crude model; Model 2: adjusted for gender, age, ethnicity, education, smoking status, and drinking status; Model 3: adjusted for gender, age, ethnicity, education, smoking status, drinking status, low-density lipoprotein cholesterol, total cholesterol, fasting blood glucose, systolic blood pressure, diastolic blood pressure, physical activity, antidiabetic agents, and diabetes duration

*HR* hazard ratio, *CI* confidence interval, *SD* standard deviation, *WC* waist circumference, *VAI* visceral adiposity index, *LAP* lipid accumulation product, *CVAI* Chinese visceral adiposity index

**Fig.3:**
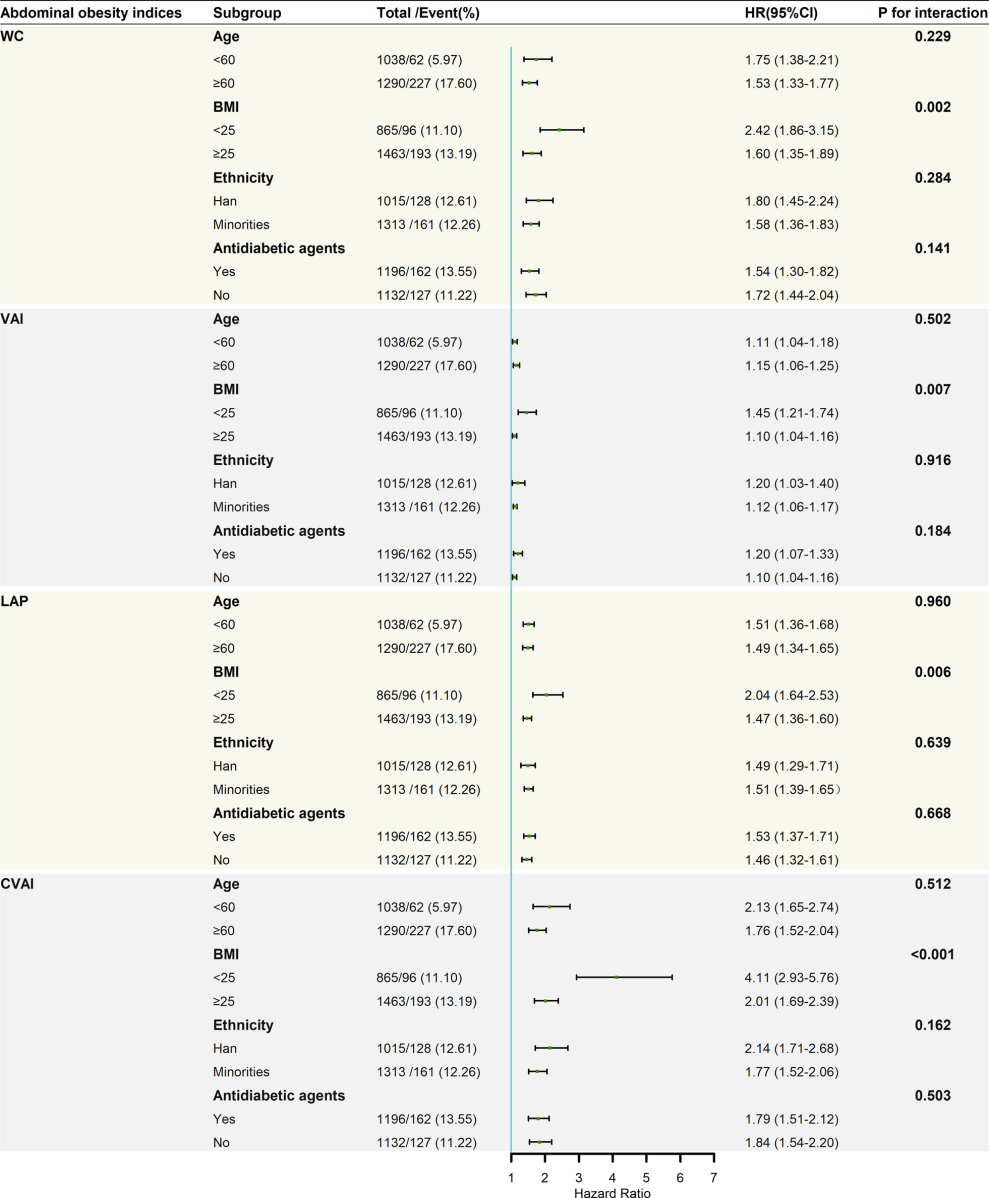
Association between abdominal obesity indices and cardiovascular events among people with type 2 diabetes in different subgroups. Each subgroup was adjusted for gender, age, ethnicity, education, smoking status, drinking status, low-density lipoprotein cholesterol, total cholesterol, fasting blood glucose, systolic blood pressure, diastolic blood pressure, physical activity, antidiabetic agents, and diabetes duration, except for stratification variables. Hazard ratios are presented as per 1 SD increase in the abdominal obesity indices for cardiovascular events. BMI, body mass index; CI, confidence interval; HR, hazard ratio; WC, waist circumference; VAI, visceral adiposity index; LAP, lipid accumulation product; CVAI, Chinese visceral adiposity index

In subgroup analyses**,** the positive correlations between abdominal obesity indices and incident CV events among people with T2D were consistent across the subgroups, including age (< 60 years vs. ≥ 60 years), ethnicity (Han vs. minorities), BMI (< 25 kg/m^2^ vs. ≥ 25 kg/m^2^), and the usage of antidiabetic agents (no vs. yes) (Fig. 3). Notably, significant interactions existed between the abdominal obesity indicators and BMI regarding the risk of CV events among people with T2D (all *P* values for interaction < 0.05). Compared to the overweight/obese group, a stronger association of each abdominal obesity index with incident CV events was found in the subgroup with BMI < 25 kg/m^2^(Fig. 3).

### Predictive performance of abdominal obesity indices for cardiovascular events in people with type 2 diabetes

Table 3 shows the predictive performance of BMI, WC, VAI, LAP, and CVAI for incident CV events in people with T2D. Our results indicated that the C-statistic of CVAI, VAI, LAP, WC, and BMI was 0.700 (95% CI 0.672–0.728), 0.656 (95% CI 0.627–0.685), 0.670 (95% CI 0.641–0.700), 0.638 (95% CI 0.608–0.668), and 0.535 (95% CI 0.501–0.569), respectively. Furthermore, CVAI had the largest C-statistic compared to VAI, LAP, WC, and BMI (all *P* for comparison < 0.05) (Table 3).

**Table 3:** Predictive performance of abdominal obesity indices for incident cardiovascular events in people with type 2 diabetes

Indices	C-statistic (95% CI)	*P*-value	*P* for comparison
CVAI	0.700 (0.672–0.728)	< 0.001	Ref.
VAI	0.656 (0.627–0.685)	< 0.001	0.012
LAP	0.670 (0.641–0.700)	< 0.001	0.035
WC	0.638 (0.608–0.668)	< 0.001	< 0.001
BMI	0.535 (0.501–0.569)	< 0.001	< 0.001

CI, confidence interval; *C-statistic* Harrell's concordance statistic, *LAP* lipid accumulation product, *VAI* visceral adiposity index, *CVAI* Chinese visceral adiposity index, *WC* waist circumference, *BMI* body mass index

### Incremental predictive value of abdominal obesity indices in the risk assessment of cardiovascular events among people with type 2 diabetes

The incremental predictive values of abdominal obesity indices for CV events are summarized in Table 4. The C-statistics of the basic model were significantly enhanced by adding WC, LAP, or CVAI (all *P* < 0.05). Moreover, after adding WC, LAP, and CVAI to the basic model, IDI showed significant improvements of 2.6% (95% CI 0.012–0.046, *P* < 0.001), 4.2% (95% CI 0.026–0.062, *P* < 0.001), and 4.3% (95% CI 0.025–0.066, *P* < 0.001) respectively. Meanwhile, NRI was also significant for WC (0.217, 95% CI 0.133–0.273, *P* < 0.001), LAP (0.306, 95% CI 0.213–0.381, *P* < 0.001), and CVAI (0.266, 95% CI 0.187–0.332, *P* < 0.001) (Table 4). When added to the basic model, VAI did not significantly improve the C-statistics (*P* = 0.169) but had a relatively small incremental impact on discrimination and reclassification ability.

**Table 4:** Improvement in discrimination and risk reclassification for cardiovascular events after adding abdominal obesity indices

Model	C-statistic (95% CI)	*P*-value	IDI (95% CI)	*P*-value	NRI (95% CI)	*P*-value
Basic model	0.701 (0.672–0.731)	Ref.	Ref.		Ref.	
+ WC	0.735 (0.708–0.762)	< 0.001	0.026 (0.012–0.046)	< 0.001	0.217 (0.133–0.273)	< 0.001
+ VAI	0.709 (0.679–0.738)	0.169	0.006 (0.001–0.013)	0.014	0.249 (0.099–0.349)	0.008
+ LAP	0.740 (0.713–0.768)	< 0.001	0.042 (0.026–0.062)	< 0.001	0.306 (0.213–0.381)	< 0.001
+ CVAI	0.751 (0.724–0.777)	< 0.001	0.043 (0.025–0.066)	< 0.001	0.266 (0.187–0.332)	< 0.001

The basic model included gender, age, ethnicity, education, smoking status, drinking status, low-density lipoprotein cholesterol, total cholesterol, fasting blood glucose, systolic blood pressure, diastolic blood pressure, physical activity, antidiabetic agents, and diabetes duration

*CI* confidence interval, *C-statistic* Harrell's concordance statistic, *IDI* integrated discrimination improvement, *NRI* net reclassification improvement, *WC* waist circumference, *VAI* visceral adiposity index, *LAP* lipid accumulation product, *CVAI* Chinese visceral adiposity index

### Sensitive analyses

In the competing risk analysis, the associations of abdominal obesity indices with CV events in people with T2D remained unchanged (Additional file 1: Fig. S2). In addition, we repeated the primary analyses after multiple imputation of missing covariates at baseline (Additional file 1: Tables S1-S3). We found that the positive correlations between abdominal obesity indices and incident CV events among people with T2D remained significant (Additional file 1: Table S1). The CVAI index had the optimal predictive capacity (C-statistic: 0.701, 95% CI 0.674–0.729, all *P* for comparison < 0.05) for CV events in people with T2D, and showed the largest incremental predictive value beyond the conventional risk factors for CV events (C-statistic: 0.752 vs. 0.704, *P* < 0.001; IDI: 4.3%, *P* < 0.001; NRI: 25.5%, *P* < 0.001) (Additional file 1: Tables S2-S3).

## Discussion

In this multiethnic cohort study, we investigated and compared the predictive performance of abdominal adiposity indices for incident CV events among people with T2D. Our results indicated that all abdominal obesity indicators were positively correlated with the risk of CV events among people with T2D. Further subgroup analysis revealed that these associations remained significant in different subgroups, including age, ethnicity, BMI, and usage of antidiabetic agents. Moreover, the CVAI index exhibited the greatest power in predicting CV events compared with BMI, WC, VAI, and LAP. When the abdominal obesity index was added to the basic risk model, the CVAI index showed the largest incremental effect on CV events risk stratification in people with T2D compared to WC, VAI, and LAP.

China has the largest obese population in the world, with more than 15% of adults suffering from overall obesity and more than 35% suffering from abdominal obesity from 2014 to 2018 [35]. Increasing evidence supported that abdominal fat accumulation was more strongly correlated with the risk of diabetes and CVD than overall obesity [36–38]. As an indicator of overall obesity, BMI fails to differentiate between muscle and fat mass, and its increase might be attributed to muscle formation rather than fat deposition from overeating [39]. Although previous studies revealed that the surrogate indicators of abdominal fat deposition have stronger predictive accuracy for CVD in the general population compared to BMI [22, 24, 40], this issue is unclear in the T2D population. Considering that people with T2D are more likely to be obese than non-T2D patients [41], studies from the general population may not be appropriate for people with T2D. Our study adds to the existing evidence indicating that abdominal adiposity indices are correlated with incident CV events and superior to BMI in predicting the risk of CV events among people with T2D. In addition, the subgroup analyses showed that WC, VAI, LAP, and CVAI had a stronger association with CV events in participants with BMI < 25 kg/m^2^ compared to overweight/obese participants. A previous study discovered that CVD mortality in adults with diabetes increased from a BMI greater than or equal to 24.8 kg/m^2^ [42], which might lead to a relatively lower additional risk of CVD caused by abdominal obesity in the overweight/obese population (BMI ≥ 25 kg/m^2^). Similarly, a prospective cohort study in Ommoord also found a stronger association of WC, VAI, and LAP with incident diabetes among non-obese individuals [19]. These data underline the importance of assessing the abdominal fat deposition in people with T2D, even among people with normal weight.

Our results indicated, among all abdominal adiposity indices, that CVAI not only had the best ability to predict incident CV events in people with T2D but also had the greatest incremental risk stratification when added to the conventional risk model. A potential explanation for these findings could be that the CVAI, constructed based on the visceral fat volume detected by computed tomography (CT) examination, is a reliable surrogate indicator to assess visceral fat mass in the Chinese population [21]. Abdominal fat accumulation includes both subcutaneous and visceral adipose tissue, of which visceral adiposity contributes more to the development of CVD than subcutaneous fat [43]. However, WC only reflects the amount of abdominal fat and fails to distinguish the amount of visceral and subcutaneous fat. LAP was established based on population frequency charts of WC and triglyceride concentrations, which could reflect central lipid accumulation and toxicity, but remained deficient in differentiating visceral from subcutaneous fat [25, 32]. In this study, although VAI was correlated with the development of CV events among people with T2D, it had a weaker predictive value for CV events than CVAI and LAP. In addition, we found that VAI did not significantly improve the C-statistic when added to the basic risk model of CV events. The VAI was designed to evaluate visceral adipose tissue in Caucasians [31], an index that combined anthropometric and metabolic measurements. Because of significant differences in adipose tissue distribution among ethnic groups [44], VAI might not accurately reflect the visceral adipose tissue of Chinese adults. Indeed, previous evidence demonstrated that VAI was inferior to CVAI, BMI, and WC in evaluating visceral adipose accumulation among Chinese adults [21].

Several underlying mechanisms were proposed to explain the association between abdominal obesity and CV events. First, macrophages often infiltrate abdominal adipose tissue, causing increased production of pro-inflammatory cytokines, such as interleukin-6 and tumor necrosis factor-α, which may induce oxidative stress and endothelial dysfunction, ultimately facilitating the development of atherosclerosis [45]. Second, visceral adipose tissue accumulation could promote changes in adipokines (leptin and adiponectin) expression, another factor contributing to inflammation and endothelial dysfunction. Available evidence indicated that leptin, a bioactive substance derived from visceral adipose tissue, could promote arterial thrombosis and vascular cell calcification [45]. Adiponectin is an anti-inflammatory adipokine, and excess visceral adipose decreases the secretion of adiponectin [46], which may enhance vascular reactive oxygen species production and impairment of endothelial function. Third, visceral adipose tissue accumulation increases the production of oxidized low-density lipoprotein (OxLDL) [45], which leads to monocyte chemotaxis, inflammation, endothelial injury, and vascular remodeling [47], thereby promoting atherosclerosis.

Some strengths in this study should be acknowledged. Primarily, our study is based on a prospective cohort of individuals with T2D, which could interpret the longitudinal associations of abdominal obesity indices with incident CV events. In addition, this study included a medium-sized, socio-economically diverse, and multiethnic Chinese population, which extends the population applicability of the findings. Finally, we adjusted for underlying confounding factors in analyses where possible, as well as performed subgroup and sensitivity analyses to guarantee the robustness of the results. However, the current study also had several limitations. First, we did not use CT and magnetic resonance imaging (MRI) to validate the consistency between the actual amount of visceral fat tissue and these abdominal obesity indices. As a result, values of abdominal obesity indices may be subject to measurement bias. Second, this observational study limits causal inferences between abdominal obesity indices and CV events in people with T2D. Third, despite adjusting for potential risk factors in multivariate analyses, some residual or unassessed confounding variables still could not be excluded. In addition, the number of outcomes events was small to establish a robust relationship between the abdominal obesity index and different subtypes of CVD, such as myocardial infarction and stroke. Finally, all participants in this study were Chinese, so our results might not be directly extendable to other populations.

## Conclusions

Overall, this prospective cohort study showed that all abdominal obesity indicators were significantly associated with the risk of CV events among people with T2D. Among these abdominal obesity indicators, CVAI exhibited the best performance for predicting incident CV events. The evidence from this study suggested that CVAI might be a valuable abdominal obesity indicator for identifying the high risk of CV events in Chinese populations with T2D. In public health practice, we could consider reducing abdominal fat deposition in people with T2D as an intervention measure to prevent CV events, particularly in people with normal weight. Large randomized clinical trials are needed to confirm the hypothesis.

## Supplementary Information

**Additional file 1: Figure S1.** Restricted cubic splines analysis of the relationship between abdominal obesity indices and the risk of cardiovascular events among people with type 2 diabetes. **Figure S2.** Subdistribution hazard ratios for the association between abdominal obesity indices and cardiovascular events in people with type 2 diabetes. **Table S1.** Association between baseline abdominal obesity indices and incident cardiovascular events in people with type 2 diabetes. **Table S2.** Predictive performance of abdominal obesity indices for incident cardiovascular events in people with type 2 diabetes. **Table S3.** Improvement in discrimination and risk reclassification for cardiovascular events after adding abdominal obesity indices. **Table S4**. Checklist of items that should be reported in cohort studies according to the STROBE statement.

## Data Availability

The datasets analysed during the current study are available from the corresponding author upon reasonable request.

## Abbreviations

T2D: Type 2 diabetes
CVD: Cardiovascular disease
BMI: Body mass index
WC: Waist circumference
VAI: Visceral adiposity index
LAP: Lipid accumulation product
CVAI: Chinese visceral adiposity index
XMC: The Xinjiang Multi-Ethnic Cohort
FBG: Fasting blood glucose
TG: Triglycerides
TC: Total cholesterol
HDL-C: High-density lipoprotein cholesterol
LDL-C: Low-density lipoprotein cholesterol
HR: Hazard ratio
CI: Confidence interval
SD: Standard deviation
C-statistic: Harrell's concordance statistic
NRI: Net reclassification improvement
IDI: Integrated discrimination improvement
SBP: Systolic blood pressure
DBP: Diastolic blood pressure
CT: Computed tomography
OxLDL: Oxidized low-density lipoprotein
MRI: Magnetic resonance imaging

## References

[1] Sun H, Saeedi P, Karuranga S, Pinkepank M, Ogurtsova K, Duncan BB, Stein C, Basit A, Chan JCN, Mbanya JC (2022). IDF diabetes Atlas: global, regional and country-level diabetes prevalence estimates for 2021 and projections for 2045. Diabetes Res Clin Pract.

[2] Shah AD, Langenberg C, Rapsomaniki E, Denaxas S, Pujades-Rodriguez M, Gale CP, Deanfield J, Smeeth L, Timmis A, Hemingway H (2015). Type 2 diabetes and incidence of cardiovascular diseases: a cohort study in 1.9 million people. Lancet Diabetes Endocrinol.

[3] Zheng Y, Ley SH, Hu FB (2018). Global aetiology and epidemiology of type 2 diabetes mellitus and its complications. Nat Rev Endocrinol.

[4] Rao Kondapally Seshasai S, Kaptoge S, Thompson A, Di Angelantonio E, Gao P, Sarwar N, Whincup PH, Mukamal KJ, Gillum RF, Holme I (2011). Diabetes mellitus, fasting glucose, and risk of cause-specific death. N Engl J Med.

[5] Einarson TR, Acs A, Ludwig C, Panton UH (2018). Prevalence of cardiovascular disease in type 2 diabetes: a systematic literature review of scientific evidence from across the world in 2007–2017. Cardiovasc Diabetol.

[6] Kivimaki M, Strandberg T, Pentti J, Nyberg ST, Frank P, Jokela M, Ervasti J, Suominen SB, Vahtera J, Sipila PN (2022). Body-mass index and risk of obesity-related complex multimorbidity: an observational multicohort study. Lancet Diabetes Endocrinol.

[7] Costanzo P, Cleland JG, Pellicori P, Clark AL, Hepburn D, Kilpatrick ES, Perrone-Filardi P, Zhang J, Atkin SL (2015). The obesity paradox in type 2 diabetes mellitus: relationship of body mass index to prognosis: a cohort study. Ann Intern Med.

[8] Zhao Y, Qie R, Han M, Huang S, Wu X, Zhang Y, Feng Y, Yang X, Li Y, Wu Y (2021). Association of BMI with cardiovascular disease incidence and mortality in patients with type 2 diabetes mellitus: a systematic review and dose-response meta-analysis of cohort studies. Nutr Metab Cardiovasc Dis.

[9] Eeg-Olofsson K, Cederholm J, Nilsson PM, Zethelius B, Nunez L, Gudbjornsdottir S, Eliasson B (2009). Risk of cardiovascular disease and mortality in overweight and obese patients with type 2 diabetes: an observational study in 13,087 patients. Diabetologia.

[10] Perotto M, Panero F, Gruden G, Fornengo P, Lorenzati B, Barutta F, Ghezzo G, Amione C, Cavallo-Perin P, Bruno G (2013). Obesity is associated with lower mortality risk in elderly diabetic subjects: the Casale Monferrato study. Acta Diabetol.

[11] Shen Y, Shi L, Nauman E, Katzmarzyk PT, Price-Haywood EG, Bazzano AN, Nigam S, Hu G (2020). Association between body mass index and stroke risk among patients with type 2 diabetes. J Clin Endocrinol Metab.

[12] Liu XM, Liu YJ, Zhan J, He QQ (2015). Overweight, obesity and risk of all-cause and cardiovascular mortality in patients with type 2 diabetes mellitus: a dose-response meta-analysis of prospective cohort studies. Eur J Epidemiol.

[13] Polemiti E, Baudry J, Kuxhaus O, Jager S, Bergmann MM, Weikert C, Schulze MB (2021). BMI and BMI change following incident type 2 diabetes and risk of microvascular and macrovascular complications: the EPIC-potsdam study. Diabetologia.

[14] Chandramouli C, Tay WT, Bamadhaj NS, Tromp J, Teng TK, Yap JJL, MacDonald MR, Hung CL, Streng K, Naik A (2019). Association of obesity with heart failure outcomes in 11 Asian regions: a cohort study. PLoS Med.

[15] Silveira EA, Kliemann N, Noll M, Sarrafzadegan N, de Oliveira C (2021). Visceral obesity and incident cancer and cardiovascular disease: an integrative review of the epidemiological evidence. Obes Rev.

[16] Koenen M, Hill MA, Cohen P, Sowers JR (2021). Obesity, adipose tissue and vascular dysfunction. Circ Res.

[17] Ross R, Neeland IJ, Yamashita S, Shai I, Seidell J, Magni P, Santos RD, Arsenault B, Cuevas A, Hu FB (2020). Waist circumference as a vital sign in clinical practice: a consensus statement from the IAS and ICCR working group on visceral obesity. Nat Rev Endocrinol.

[18] Wilding JPH, Jacob S (2021). Cardiovascular outcome trials in obesity: a review. Obes Rev.

[19] Brahimaj A, Rivadeneira F, Muka T, Sijbrands EJG, Franco OH, Dehghan A, Kavousi M (2019). Novel metabolic indices and incident type 2 diabetes among women and men: the Rotterdam study. Diabetologia.

[20] Sun K, Lin D, Feng Q, Li F, Qi Y, Feng W, Yang C, Yan L, Ren M, Liu D (2019). Assessment of adiposity distribution and its association with diabetes and insulin resistance: a population-based study. Diabetol Metab Syndr.

[21] Xia MF, Chen Y, Lin HD, Ma H, Li XM, Aleteng Q, Li Q, Wang D, Hu Y, Pan BS (2016). A indicator of visceral adipose dysfunction to evaluate metabolic health in adult Chinese. Sci Rep.

[22] Kyrou I, Panagiotakos DB, Kouli GM, Georgousopoulou E, Chrysohoou C, Tsigos C, Tousoulis D, Pitsavos C (2018). Lipid accumulation product in relation to 10-year cardiovascular disease incidence in Caucasian adults: the ATTICA study. Atherosclerosis.

[23] Kouli GM, Panagiotakos DB, Kyrou I, Georgousopoulou EN, Chrysohoou C, Tsigos C, Tousoulis D, Pitsavos C (2017). Visceral adiposity index and 10-year cardiovascular disease incidence: the ATTICA study. Nutr Metab Cardiovasc Dis.

[24] Wang L, Lee Y, Wu Y, Zhang X, Jin C, Huang Z, Wang Y, Wang Z, Kris-Etherton P, Wu S (2021). A prospective study of waist circumference trajectories and incident cardiovascular disease in China: the Kailuan Cohort study. Am J Clin Nutr.

[25] Wan H, Wang Y, Xiang Q, Fang S, Chen Y, Chen C, Zhang W, Zhang H, Xia F, Wang N (2020). Associations between abdominal obesity indices and diabetic complications: Chinese visceral adiposity index and neck circumference. Cardiovasc Diabetol.

[26] Hosseinpanah F, Barzin M, Mirbolouk M, Abtahi H, Cheraghi L, Azizi F (2016). Lipid accumulation product and incident cardiovascular events in a normal weight population: Tehran Lipid and Glucose Study. Eur J Prev Cardiol.

[27] Yi X, Ling J, Meng H, Wu L, Zhu S, Zhu L (2022). Lipid accumulation product predicts diabetes remission after bariatric surgery in Chinese patients with BMI < 35 kg/m(2): a Multicenter Cohort Study. Obes Surg.

[28] Zhang X, Hong F, Liu L, Nie F, Du L, Guan H, Wang Z, Zeng Q, Yang J, Wang J (2022). Lipid accumulation product is a reliable indicator for identifying metabolic syndrome: the China Multi-Ethnic Cohort (CMEC) Study. QJM.

[29] Tao L, Tian T, Liu L, Zhang Z, Sun Q, Sun G, Dai J, Yan H (2022). Cohort profile: the Xinjiang multiethnic cohort (XMC) study. BMJ Open.

[30] Choi Y, Choi JW (2020). Association of sleep disturbance with risk of cardiovascular disease and all-cause mortality in patients with new-onset type 2 diabetes: data from the Korean NHIS-HEALS. Cardiovasc Diabetol.

[31] Amato MC, Giordano C, Galia M, Criscimanna A, Vitabile S, Midiri M, Galluzzo A, AlkaMeSy Study G (2010). Visceral adiposity index: a reliable indicator of visceral fat function associated with cardiometabolic risk. Diabetes Care..

[32] Kahn HS (2005). The "lipid accumulation product" performs better than the body mass index for recognizing cardiovascular risk: a population-based comparison. BMC Cardiovasc Disord.

[33] Guasch-Ferre M, Liu G, Li Y, Sampson L, Manson JE, Salas-Salvado J, Martinez-Gonzalez MA, Stampfer MJ, Willett WC, Sun Q (2020). Olive oil consumption and cardiovascular risk in U.S Adults. J Am Coll Cardiol.

[34] DeLong ER, DeLong DM, Clarke-Pearson DL (1988). Comparing the areas under two or more correlated receiver operating characteristic curves: a nonparametric approach. Biometrics.

[35] Mu L, Liu J, Zhou G, Wu C, Chen B, Lu Y, Lu J, Yan X, Zhu Z, Nasir K (2021). Obesity prevalence and risks among Chinese adults: findings from the China PEACE million persons project, 2014–2018. Circ Cardiovasc Qual Outcomes.

[36] Cornier MA, Despres JP, Davis N, Grossniklaus DA, Klein S, Lamarche B, Lopez-Jimenez F, Rao G, St-Onge MP, Towfighi A (2011). Assessing adiposity: a scientific statement from the American heart association. Circulation.

[37] Nyamdorj R, Qiao Q, Lam TH, Tuomilehto J, Ho SY, Pitkaniemi J, Nakagami T, Mohan V, Janus ED, Decoda Study G (2008). BMI compared with central obesity indicators in relation to diabetes and hypertension in Asians. Obesity (Silver Spring).

[38] Yusuf S, Hawken S, Ounpuu S, Bautista L, Franzosi MG, Commerford P, Lang CC, Rumboldt Z, Onen CL, Lisheng L (2005). Obesity and the risk of myocardial infarction in 27,000 participants from 52 countries: a case-control study. Lancet.

[39] Nevill AM, Stewart AD, Olds T, Holder R (2006). Relationship between adiposity and body size reveals limitations of BMI. Am J Phys Anthropol.

[40] Huang YC, Huang JC, Lin CI, Chien HH, Lin YY, Wang CL, Liang FW, Dai CY, Chuang HY (2021). Comparison of innovative and traditional cardiometabolic indices in estimating atherosclerotic cardiovascular disease risk in adults. Diagnostics (Basel).

[41] Xing Z, Pei J, Huang J, Peng X, Chen P, Hu X (2018). Relationship of obesity to adverse events among patients with mean 10-year history of type 2 diabetes mellitus: results of the ACCORD Study. J Am Heart Assoc.

[42] Dahlstrom EH, Sandholm N, Forsblom CM, Thorn LM, Jansson FJ, Harjutsalo V, Groop PH (2019). Body mass index and mortality in individuals with type 1 diabetes. J Clin Endocrinol Metab.

[43] Powell-Wiley TM, Poirier P, Burke LE, Despres JP, Gordon-Larsen P, Lavie CJ, Lear SA, Ndumele CE, Neeland IJ, Sanders P (2021). Obesity and cardiovascular disease: a scientific statement from the American heart association. Circulation.

[44] Chirinos DA, Llabre MM, Goldberg R, Gellman M, Mendez A, Cai J, Sotres-Alvarez D, Daviglus M, Gallo LC, Schneiderman N (2020). Defining abdominal obesity as a risk factor for coronary heart disease in the U.S: results from the hispanic community health study/study of Latinos (HCHS/SOL). Diabetes Care.

[45] Van Gaal LF, Mertens IL, De Block CE (2006). Mechanisms linking obesity with cardiovascular disease. Nature.

[46] Mathieu P, Lemieux I, Despres JP (2010). Obesity, inflammation, and cardiovascular risk. Clin Pharmacol Ther.

[47] Couillard C, Ruel G, Archer WR, Pomerleau S, Bergeron J, Couture P, Lamarche B, Bergeron N (2005). Circulating levels of oxidative stress markers and endothelial adhesion molecules in men with abdominal obesity. J Clin Endocrinol Metab.

