# Supplementary material for: Association between abdominal obesity indices and risk of cardiovascular events in Chinese populations with type 2 diabetes: a prospective cohort study
Source: Cardiovasc Diabetol. 2022 Nov 1;21:225. doi: 10.1186/s12933-022-01670-x (PMC9628026; doi:10.1186/s12933-022-01670-x)
Supplement: Supplementary file 1 — Additional file 1: Figure S1. Restricted cubic splines analysis of the relationship between abdominal obesity indices and the risk of cardiovascular events among people with type 2 diabetes. Figure S2. Subdistribution hazard ratios for the association between abdominal obesity indices and cardiovascular events in people with type 2 diabetes. Table S1. Association between baseline abdominal obesity indices and incident cardiovascular events in people with type 2 diabetes. Table S2. Predictive performance of abdominal obesity indices for incident cardiovascular events in people with type 2 diabetes. Table S3. Improvement in discrimination and risk reclassification for cardiovascular events after adding abdominal obesity indices. Table S4. Checklist of items that should be reported in cohort studies according to the STROBE statement. [file 12933_2022_1670_MOESM1_ESM.docx]

**Supplementary Material**

**
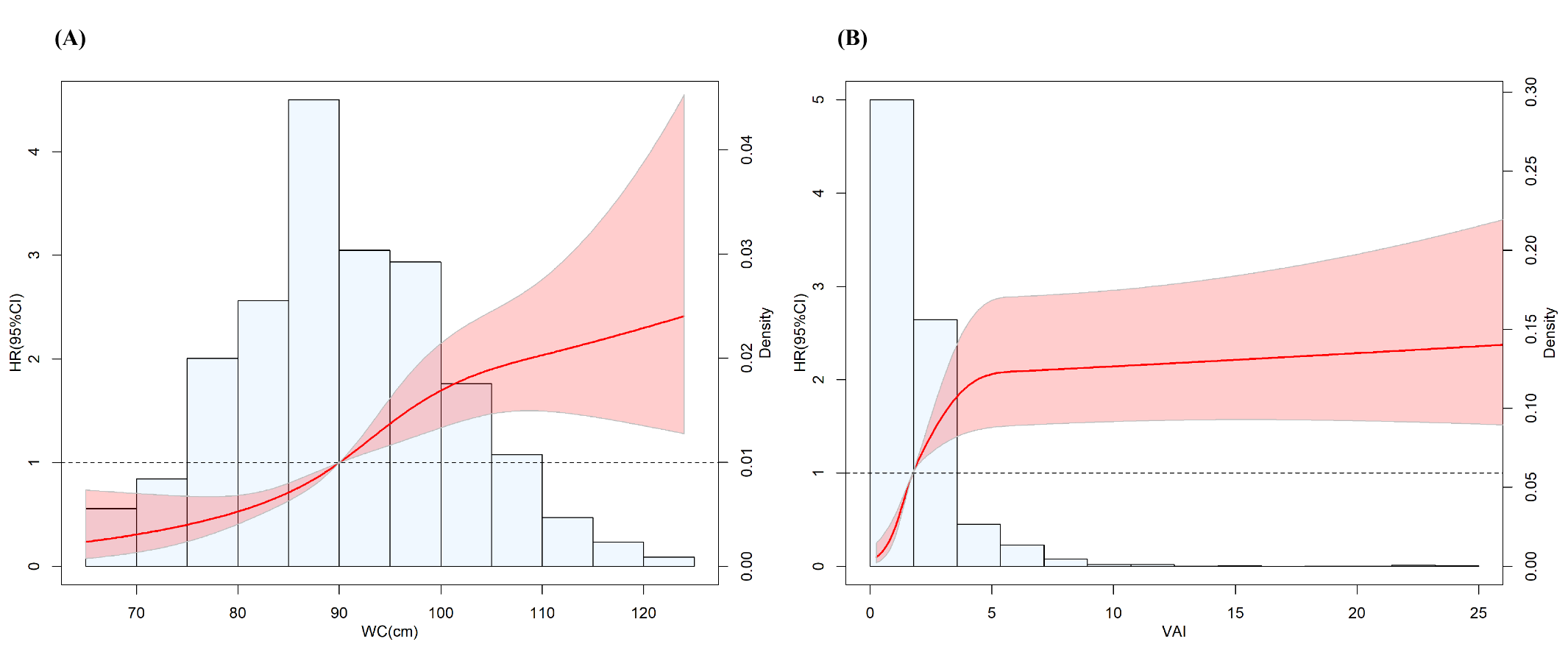
**

**
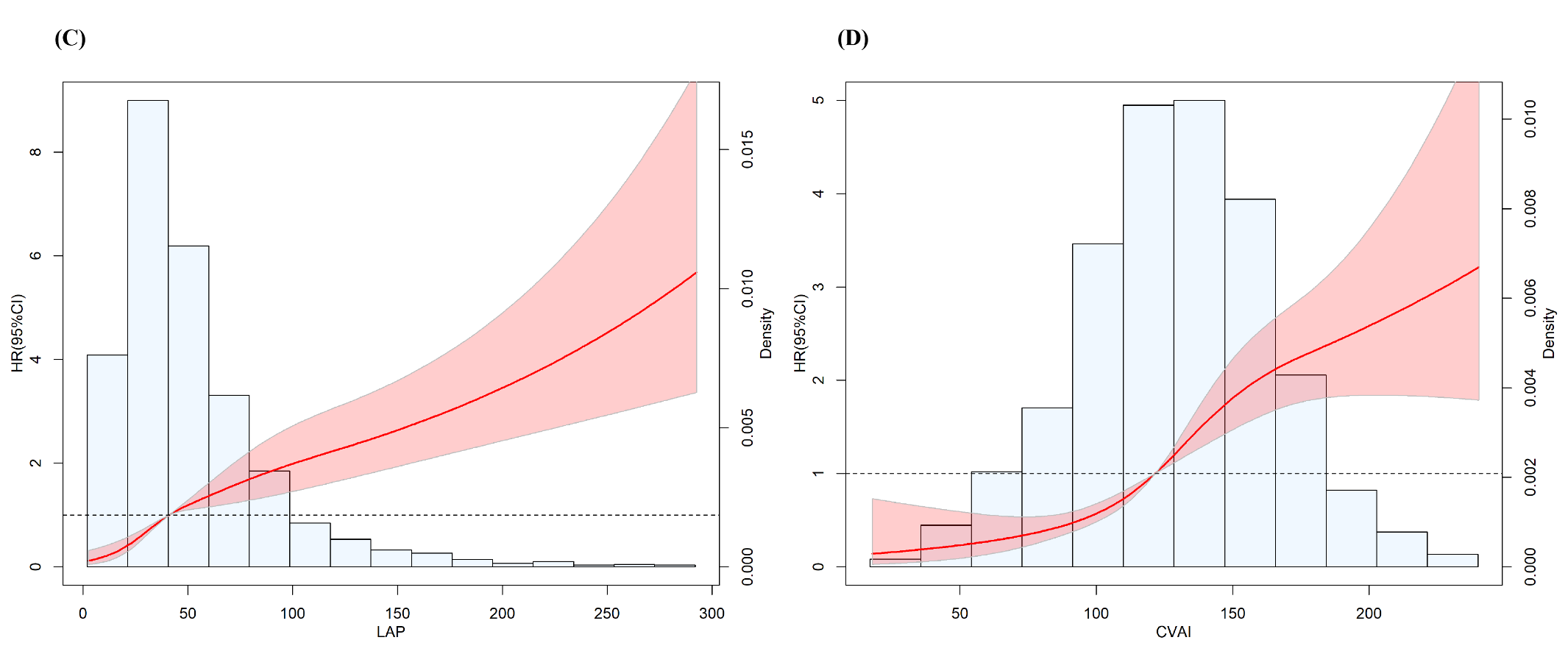
**

**Figure S1.** Restricted cubic splines analysis of the relationship between abdominal obesity indices and the risk of cardiovascular events among people with type 2 diabetes. **(A)** waist circumference (WC); **(B)** visceral adiposity index (VAI); **(C)** lipid accumulation product (LAP); **(D)** Chinese visceral adiposity index (CVAI). All abdominal obesity indices were assessed as continuous variables using restricted cubic spline regression, adjusted for gender, age, ethnicity, education, smoking status, drinking status, low-density lipoprotein cholesterol, total cholesterol, fasting blood glucose, systolic blood pressure, diastolic blood pressure, physical activity, antidiabetic agents, and diabetes duration. HR, hazard ratio; CI, confidence interval.

**
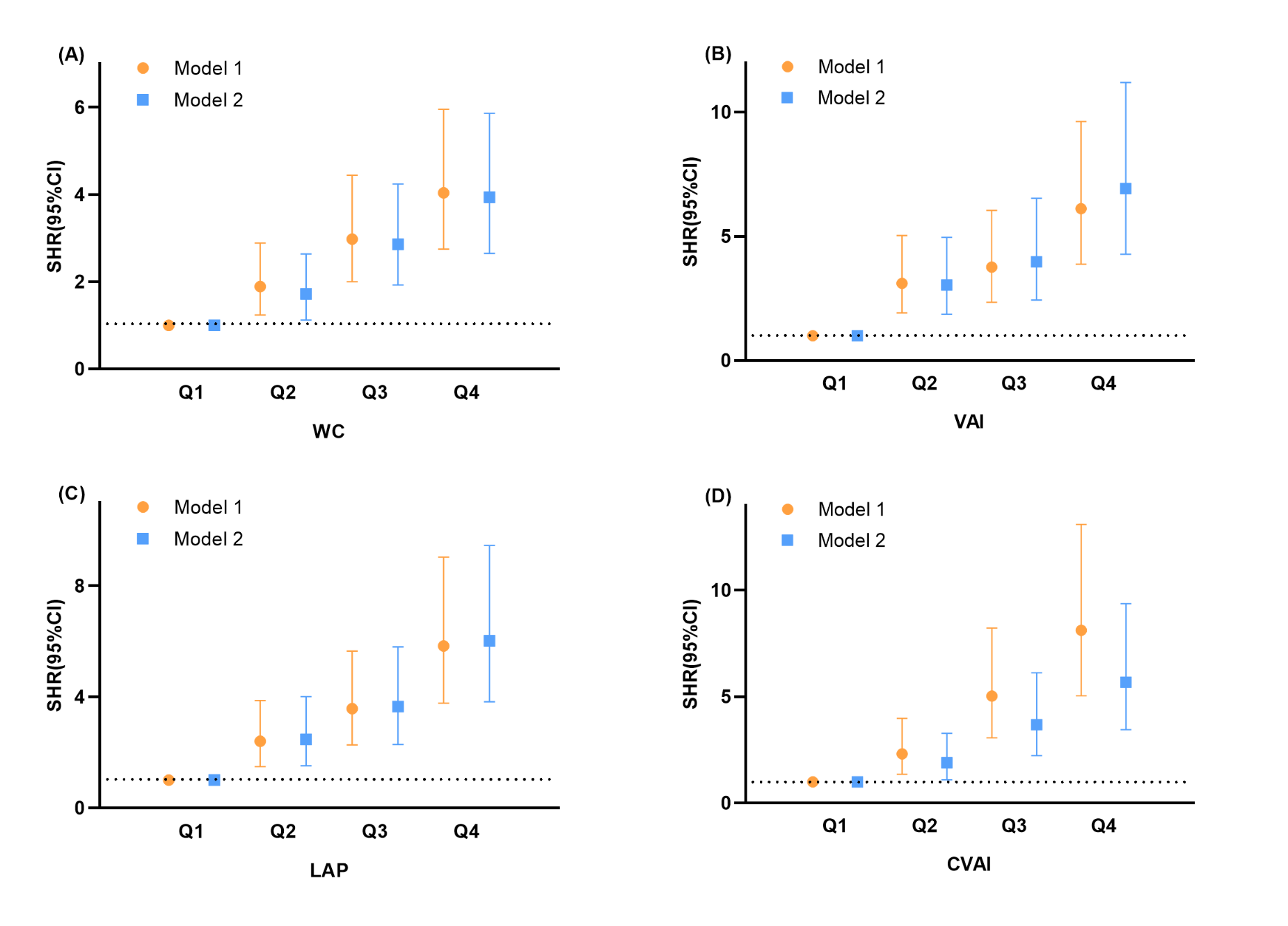
**

**Figure S2.** Subdistribution hazard ratios for the association between abdominal obesity indices and cardiovascular events in people with type 2 diabetes. Model 1: crude model. Model 2: adjusted for gender, age, ethnicity, education, smoking status, drinking status, low-density lipoprotein cholesterol, total cholesterol, fasting blood glucose, systolic blood pressure, diastolic blood pressure, physical activity, antidiabetic agents, and diabetes duration. **(A)** waist circumference (WC); **(B)** visceral adiposity index (VAI); **(C)** lipid accumulation product (LAP); **(D)** Chinese visceral adiposity index (CVAI). SHR, subdistribution hazard ratio; CI, confidence interval.

**Table S1.** Association between baseline abdominal obesity indices and incident cardiovascular events in people with type 2 diabetes*

| **Indices** | **Event/**  **Total** | **Model 1** | | **Model 2** | | **Model 3** | |
| --- | --- | --- | --- | --- | --- | --- | --- |
|  |  | ***HR* (95%CI)** | ***P*-value** | ***HR* (95%CI)** | ***P*-value** | ***HR* (95%CI)** | ***P*-value** |
| **WC** |  |  |  |  |  |  |  |
| Per 1 SD increase | 295/2442 | 1.54 (1.38-1.71) | <0.001 | 1.61 (1.42-1.81) | <0.001 | 1.58 (1.40-1.78) | <0.001 |
| **VAI** |  |  |  |  |  |  |  |
| Per 1 SD increase | 295/2442 | 1.09 (1.05-1.13) | <0.001 | 1.11 (1.07-1.16) | <0.001 | 1.11 (1.06-1.16) | <0.001 |
| **LAP** |  |  |  |  |  |  |  |
| Per 1 SD increase | 295/2442 | 1.40 (1.31-1.49) | <0.001 | 1.47 (1.37-1.57) | <0.001 | 1.46 (1.36-1.57) | <0.001 |
| **CVAI** |  |  |  |  |  |  |  |
| Per 1 SD increase | 295/2442 | 1.89 (1.70-2.10) | <0.001 | 1.81 (1.60-2.04) | <0.001 | 1.78 (1.57-2.01) | <0.001 |

* Results of the sensitivity analyses after multiple imputation of missing covariates at baseline. Model 1: crude model; Model 2: adjusted for gender, age, ethnicity, education, smoking status, and drinking status; Model 3: adjusted for gender, age, ethnicity, education, smoking status, drinking status, low-density lipoprotein cholesterol, total cholesterol, fasting blood glucose, systolic blood pressure, diastolic blood pressure, physical activity, antidiabetic agents, and diabetes duration. HR, hazard ratio; CI, confidence interval; SD, standard deviation; WC, waist circumference; VAI, visceral adiposity index; LAP, lipid accumulation product; CVAI, Chinese visceral adiposity index.

**Table S2.** Predictive performance of abdominal obesity indices for incident cardiovascular events in people with type 2 diabetes*

| **Indices** | **C-****statistic (95%CI)** | ***P*-value** | ***P* for comparison** |
| --- | --- | --- | --- |
| CVAI | 0.701 (0.674-0.729) | <0.001 | Ref. |
| VAI | 0.653 (0.624-0.682) | <0.001 | 0.015 |
| LAP | 0.669 (0.639-0.698) | <0.001 | 0.004 |
| WC | 0.640 (0.610-0.670) | <0.001 | <0.001 |
| BMI | 0.539 (0.506-0.573) | <0.001 | <0.001 |

* Results of the sensitivity analyses after multiple imputation of missing covariates at baseline. CI, confidence interval; C-statistic, Harrell's concordance statistic; LAP, lipid accumulation product; VAI, visceral adiposity index; CVAI, Chinese visceral adiposity index; WC, waist circumference; BMI, body mass index.

**Table S3.** Improvement in discrimination and risk reclassification for cardiovascular events after adding abdominal obesity indices*

| **Model** | **C-****statistic**  **(95%CI)** | ***P*-value** | **IDI**  **(95%CI)** | ***P*-value** | **NRI**  **(95%CI)** | ***P*-value** |
| --- | --- | --- | --- | --- | --- | --- |
| Basic model | 0.704 (0.675-0.773) | Ref. | Ref. |  | Ref. |  |
| +WC | 0.738 (0.711-0.765) | <0.001 | 0.027 (0.014-0.046) | <0.001 | 0.211 (0.133-0.274) | <0.001 |
| +VAI | 0.711 (0.682-0.740) | 0.078 | 0.005 (0.001-0.014) | 0.012 | 0.245 (0.089-0.332) | 0.008 |
| +LAP | 0.742 (0.715-0.770) | <0.001 | 0.040 (0.025-0.059) | <0.001 | 0.314 (0.225-0.374) | <0.001 |
| +CVAI | 0.752 (0.727-0.778) | <0.001 | 0.043 (0.025-0.065) | <0.001 | 0.255 (0.189-0.323) | <0.001 |

* Results of the sensitivity analyses after multiple imputation of missing covariates at baseline. The basic model included gender, age, ethnicity, education, smoking status, drinking status, low-density lipoprotein cholesterol, total cholesterol, fasting blood glucose, systolic blood pressure, diastolic blood pressure, physical activity, antidiabetic agents, and diabetes duration. CI, confidence interval; C-statistic, Harrell's concordance statistic; IDI, integrated discrimination improvement; NRI, net reclassification improvement; WC, waist circumference; VAI, visceral adiposity index; LAP, lipid accumulation product; CVAI, Chinese visceral adiposity index.

**Table S4.** Checklist of items that should be reported in cohort studies according to the STROBE statement.

|  | **Item No.** | **STROBE items** | **Location in the manuscript** |
| --- | --- | --- | --- |
| **Title and abstract** |  |  |  |
|  | 1 | (a) Indicate the study's design with a commonly used term in the title or the abstract  (b) Provide in the abstract an informative and balanced summary of what was done and what was found | Page 1-3 |
| **Introduction** |  |  |  |
| Background/rationale | 2 | Explain the scientific background and rationale for the investigation being reported | Page 3-5 |
| Objectives | 3 | State specific objectives, including any prespecified hypotheses | Page 5 |
| **Methods** |  |  |  |
| Study design | 4 | Present key elements of study design early in the paper | Page 5-6 |
| Setting | 5 | Describe the setting, locations, and relevant dates, including periods of recruitment, exposure, follow-up, and data collection | Page 5-8 |
| Participants | 6 | (a) Give the eligibility criteria, and the sources and methods of selection of participants. Describe methods of follow-up  (b) For matched studies, give matching criteria and number of exposed and unexposed | Page 5-8 |
| Variables | 7 | Clearly define all outcomes, exposures, predictors, potential confounders, and effect modifiers. Give diagnostic criteria, if applicable | Page 6-8 |
| Data sources/ measurement | 8 | For each variable of interest, give sources of data and details of methods of assessment (measurement). Describe comparability of assessment methods if there is more than one group | Page 6-8 |
| Bias | 9 | Describe any efforts to address potential sources of bias | Page 5-8 |
| Study size | 10 | Explain how the study size was arrived at | Page 5-6 |
| Quantitative variables | 11 | Explain how quantitative variables were handled in the analyses. If applicable, describe which groupings were chosen and why | Page 6-8 |
| Statistical methods | 12 | (a) Describe all statistical methods, including those used to control for confounding  (b) Describe any methods used to examine subgroups and interactions  (c) Explain how missing data were addressed  (d) If applicable, explain how loss to follow-up was addressed  (e) Describe any sensitivity analyses | Page 8-10 |
| **Results** |  |  |  |
| Participants | 13 | (a) Report the numbers of individuals at each stage of the study -*e.g.*, numbers potentially eligible, examined for eligibility, confirmed eligible, included in the study, completing follow-up, and analysed  (b) Give reasons for non-participation at each stage.  (c) Consider use of a flow diagram | a) Page 5-6  c) Figure 1 |
| Descriptive data | 14 | (a) Give characteristics of study participants (*e.g.*, demographic, clinical, social) and information on exposures and potential confounders  (b) Indicate the number of participants with missing data for each variable of interest  (c) Summarise follow-up time (*e.g.*, average and total amount) | a) Page10-11  b) Page 5-6  c) Page 10 |
| Outcome data | 15 | Report numbers of outcome events or summary measures over time | Page 10 |
| Main results | 16 | (a) Give unadjusted estimates and, if applicable, confounder-adjusted estimates and their precision (e.g., 95% confidence interval). Make clear which confounders were adjusted for and why they were included  (b) Report category boundaries when continuous variables were categorized  (c) If relevant, consider translating estimates of relative risk into absolute risk for a meaningful time period | Page 11-13 |
| Other analyses | 17 | Report other analyses done—e.g., analyses of subgroups and interactions, and sensitivity analyses | Page 13-17 and supplementary files (Figure 2, Tables S1-S3) |
| **Discussion** |  |  |  |
| Key results | 18 | Summarise key results with reference to study objectives | Page 18 |
| Limitations | 19 | Discuss limitations of the study, taking into account sources of potential bias or imprecision. Discuss both direction and magnitude of any potential bias | Page 21-22 |
| Interpretation | 20 | Give a cautious overall interpretation of results considering objectives, limitations, multiplicity of analyses, results from similar studies, and other relevant evidence | Page 18-21 |
| Generalisability | 21 | Discuss the generalisability (external validity) of the study results | Page 18-21 |
| **Other Information** |  |  |  |
| Funding | 22 | Give the source of funding and the role of the funders for the present study and, if applicable, for the original study on which the present article is based | Page 24 |

* von Elm E, Altman DG, Egger M, Pocock SJ, Gøtzsche PC, Vandenbroucke JP; STROBE Initiative. The Strengthening the Reporting of Observational Studies in Epidemiology (STROBE)statement: guidelines for reporting observational studies. Lancet. 2007;370(9596):1453-7.
